# Smartphone-based real-time feedback to suppress trunk compensation for unsupervised upper limb rehabilitation in patients with brain disorders

**DOI:** 10.1186/s12984-026-02093-5

**Published:** 2026-07-16

**Authors:** Su Hyeon Kim, Doo Na Cho, Won Hyuk Chang, Hyo-Chang Seo

**Affiliations:** 1 Digital Therapeutics Research Center, Smart Healthcare Research Institute, Samsung Medical Center, Seoul, 06351 South Korea; 2 Department of Health Sciences and Technology, SAIHST, Sungkyunkwan University, Seoul, 06355 Korea; 3 Department of Medical Device Management and Research, SAIHST, Sungkyunkwan University, Seoul, 06355 Korea; 4 Department of Digital Healthcare, SAIHST, Sungkyunkwan University, Seoul, 06355 Korea; 5 Department of Physical and Rehabilitation Medicine, Center for Prevention and Rehabilitation, Heart Vascular Stroke Institute, Samsung Medical Center, Sungkyunkwan University School of Medicine, Seoul, South Korea

**Keywords:** Compensatory trunk movement, Real-time feedback, Smartphone-based system, Trunk control, Unsupervised upper limb rehabilitation

## Abstract

**Background:**

To ensure continuous rehabilitation for patients with hemiparesis due to brain disorders, there is a growing need for simple, accessible systems that enable unsupervised self-training. The occurrence of compensatory trunk movements during the unsupervised exercise may prevent true functional recovery. This study proposes a smartphone-based visual-auditory feedback system designed to detect and suppress compensatory trunk movements in real-time without wearable sensors.

**Methods:**

A pilot cohort (n = 16) was first used to calibrate detection thresholds for compensatory trunk movements. Subsequently, a total of 55 hemiparetic patients were enrolled in a randomized controlled trial and allocated to a Feedback (FB) group (n = 27) or a Non-Feedback (NFB) group (n = 28). Participants performed standardised upper limb rehabilitation tasks using the Rapael Smart Board™, a planar upper limb rehabilitation device. The proposed system utilised a smartphone camera with MediaPipe-based pose estimation to track trunk movements and provided real-time traffic-light feedback based on calibrated thresholds. Outcome measures included trunk path length, trunk deviation, task efficiency, and spatial occupancy, which were evaluated using 3D coordinates reconstructed from depth camera data.

**Results:**

The FB group demonstrated significantly improved postural stability compared to the NFB group, with a 37.9% reduction in trunk path length (*p* = 0.014) and a 35.3% decrease in spatial occupancy (*p* = 0.003). Kinematic analysis revealed that the NFB group's shorter hand path lengths were achieved through kinematic redundancy—specifically, the recruitment of trunk degrees of freedom—rather than through selective upper limb motor control. In contrast, the FB group maintained a stable posture near the neutral position, ensuring true upper limb engagement. The usability assessment demonstrated that the system was well received by users and was reliable (Cronbach's *α *= 0.775).

**Conclusion:**

The proposed system, which integrates mobile technology, effectively suppresses compensatory trunk movements and promotes selective motor control, and ensures that rehabilitation outcomes reflect true upper limb joint engagement rather than kinematic redundancy through compensatory trunk recruitment. While certain design considerations remain, particularly related to dynamic recalibration and the use of a fixed auditory feedback window, the system retains strong potential as an automated feedback solution, offering a scalable and accessible pathway for high-quality unsupervised home rehabilitation.

**Supplementary Information:**

The online version contains supplementary material available at https://doi.org/10.1186/s12984-026-02093-5.

## Background

The global prevalence of brain disorders, including stroke, brain tumours, and neurodegenerative diseases, is gradually increasing due to ageing populations, creating a significant challenge for healthcare systems worldwide [1, 2]. Among the various sequelae, upper limb hemiparesis is one of the most common and persistent disabilities, affecting a significant proportion of survivors [3, 4]. The existing clinical evidence indicates that intensive and repetitive motor training is the most significant factor in achieving functional recovery [5, 6]. However, despite this clinical importance, many patients are unable to receive sufficient rehabilitation due to limited accessibility, high costs, and a shortage of rehabilitation resources [7]. Patients are often required to perform exercises independently in unsupervised settings, where the lack of expert guidance often makes it difficult to maintain proper rehabilitation quality [8, 9]. In upper limb rehabilitation, particularly when shoulder and elbow control is impaired, patients frequently rely on compensatory trunk movements, such as forward or lateral leaning, to accomplish tasks [10, 11]. Although this compensation allows for immediate task completion, such movements can disturb functional recovery and lead to poorer long-term rehabilitation outcomes [12, 13]. Therefore, objective monitoring and management of trunk compensation are essential for effective rehabilitation.

Conventionally, therapists employed interventions to rectify compensatory postures through the utilisation of verbal cues or physical restraints [14, 15]. However, such experiential approaches are labour-intensive and often rely heavily on therapist expertise, resulting in variability in care quality across clinical settings [16, 17]. Given the limited number of rehabilitation professionals, it is difficult to provide continuous one-to-one supervision for all patients, particularly in long-term or home-based settings [18]. These challenges highlight the need for automated rehabilitation systems that can monitor patient movement and provide real-time feedback. Such systems capable of autonomously detecting trunk compensation and delivering real-time feedback have the potential to reduce therapist workload while enabling effective, scalable, and unsupervised rehabilitation in home settings where professional oversight is limited.

To facilitate motor training, a variety of technology-aided systems developed, which may be broaadly categorized into robotic and sensor-based approaches [19, 20]. Robotic systems, including exoskeletons such as the ArmeoPower (Hocoma AG, Switzerland) [21] and end-effector devices such as the InMotion ARM (Bionik Laboratories (Bionik Labs), USA) [22, 23], offer precise kinematic guidance and high-intensity training [24]. However, despite their clinical efficacy, these systems are not well suited for home use. These devices are bulky and costly, pose safety risks in unsupervised environments, and require complex setups that depend on therapist assistance [25, 26]. Wearable sensor systems, such as ArmeoSenso (Hocoma AG, Switzerland), allow motion tracking without restricting movement, but they require multiple sensors to be attached to the body, which can be challenging for patients with hemiparesis [27–29]. Conversely, passive mobile arm devices, such as smart boards, connected to a personal computer offer a practical and non-invasive alternative. These devices are cost-effective, simple to install, and safe for unsupervised use, thus making them ideal for home-based rehabilitation [30, 31]. However, a key limitation of these end-effector systems is the lack of proximal body monitoring. The system's emphasis on hand trajectory results in a lack of consideration for the user's posture. Consequently, patients frequently achieve task goals unconsciously by leaning their trunk forward rather than extending their arm as compensation. This underscores the necessity for a solution that preserves the simplicity of the Smart Board while incorporating a supervisory role to detect and correct trunk compensation. Early approaches primarily relied on the use of wearable inertial measurement units (IMUs), motion capture systems, and depth-sensing cameras such as the Microsoft Kinect and Intel RealSense to detect trunk lean [32–35]. Although IMU and motion capture-based systems facilitate quantitative analysis, the calibration and configuration of such setups are often complex and physically inconvenient for patients, making them unrealistic for unsupervised environments [36, 37].

Recent advancements in deep learning-based computer vision represent a paradigm shift towards marker-less pose estimation. It was demonstrated that frameworks such as Google's MediaPipe have the capacity to track body landmarks using only image input from standard RGB cameras [38]. Previous studies comparing MediaPipe to optical motion capture systems have reported reasonable performance and strong correlations in joint trajectories [39, 40]. This suggests that a standard smartphone camera can now serve as an accessible, contactless monitoring tool to complement simple rehabilitation devices, offering a promising avenue for scalable, unsupervised home rehabilitation. Several markerless approaches have been explored for detecting compensatory trunk movements during upper limb rehabilitation, including depth-sensing camera systems [34], RGB-based body tracking for trunk flexion monitoring in robotic rehabilitation [41], and contactless joint angle estimation during shoulder exercises [42]. A systematic review further confirmed the growing applicability of markerless motion capture in clinical rehabilitation settings [43]. However, these systems have predominantly been validated in controlled laboratory environments and rely on fixed camera setups or dedicated hardware, limiting their scalability for unsupervised home use. To date, no study has integrated real-time threshold-based feedback for trunk compensation suppression using a standard smartphone camera alone, highlighting a clear gap that the present study aims to address.

In this study, an integrated rehabilitation system is proposed, combining the accessibility of the Neofect Smart Board™ (Neofect Co., Ltd, South Korea), a planar upper limb rehabilitation device designed for task-oriented arm training [44], with the monitoring capability of a smartphone. Conventional one-to-one occupational therapy allows therapists to tailor interventions according to individual patient needs, which can enhance clinical effectiveness. However, such personalised therapy also introduces variability in task execution, making it difficult to standardise movement conditions across participants. In contrast, the Smart Board enables patients to perform identical task-oriented exercises within a controlled planar workspace, thereby reducing inter-subject variability. Because the primary objective of this study was to evaluate whether appropriate feedback could suppress compensatory trunk movements during task execution, it was important for all participants to perform standardised tasks. Therefore, the Smart Board was adopted as the training platform to ensure consistent task conditions across participants. While simple training devices often lack the supervision necessary for quality movement, we augmented the Smart Board setup with a mobile-based visual-auditory feedback system. The system utilises a standard smartphone camera and MediaPipe-based pose estimation to monitor trunk movements in real time. To ensure objectivity, we employed a data-driven thresholding strategy to categorize posture deviations, moving away from subjective manual assessments. We hypothesised that this multimodal automated feedback system will empower patients to self-correct their movements, effectively mitigating compensatory trunk patterns during unsupervised rehabilitation.

## Methods

### Participants

This study was conducted between May 2024 and September 2025. Patients with upper limb hemiparesis resulting from brain disorders such as stroke and brain tumours were recruited from the Department of Physical and Rehabilitation Medicine, Samsung Medical Center, Seoul, Republic of Korea. The affected limb was defined as the limb contralateral to the brain lesion and was not necessarily the dominant limb. Inclusion criteria were as follows: a confirmed diagnosis of a brain disorder with hemiparesis, ability to maintain a sitting posture, and a Korean Mini-Mental State Examination-2 (K-MMSE-2) score of 18 or higher is required to exclude moderate to severe cognitive impairment (scores 0–17) [45, 46]. Exclusion criteria encompassed severe upper limb spasticity or the inability to perform rehabilitation tasks independently.

**Fig. 1:**
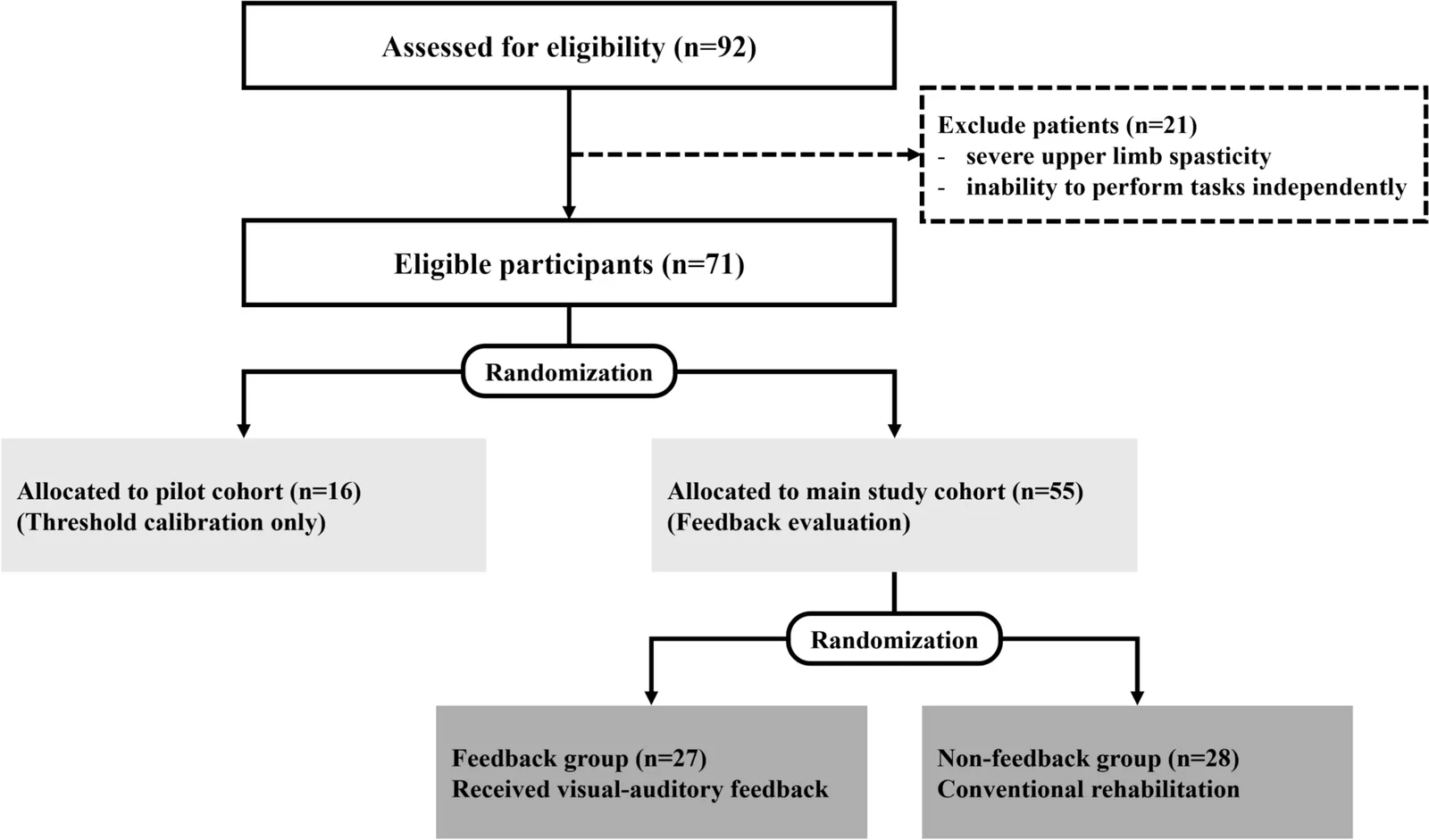
Participants cohort. Among enrolled participants, an initial pilot cohort was used for calibration of feedback thresholds, and the remaining main cohort was randomized into FB and NFB groups for outcome analyses

Initially, 92 participants were assessed for eligibility. Following a clinical assessment by a single rehabilitation specialist, 21 participants were excluded due to severe upper limb spasticity or inability to perform tasks independently, resulting in 71 eligible participants (Fig. 1).

Of the 71 eligible participants, 16 were randomly selected using a computer-generated random number sequence to form the pilot cohort for system calibration and threshold determination. The pilot cohort comprised 8 males (50.0%) and 8 females (50.0%) and was not included in the primary comparative analysis. To verify the representativeness of the pilot cohort, demographic and baseline clinical characteristics were compared between the pilot and main study cohorts. No statistically significant differences were found in any variable (all p > 0.05), confirming that the pilot cohort was representative of the overall study population in Table 1.

**Table 1:** Demographic and clinical characteristics of the overall cohort (n = 71)

	Pilot cohort (N = 16)	Main cohort (N = 55)	*p*-value
Demographics			
Age (years)	54.1 ± 17.9	55.6 ± 16.6	0.767
Gender, n (%)			1.000
Male	8 (50.0%)	28 (50.9%)	
Female	8 (50.0%)	27 (49.1%)	
Affected side, n (%)			0.575
Right	10 (62.5%)	29 (52.7%)	
Left	6 (37.5%)	26 (47.3%)	
Diagnosis, n (%)			0.110
Stroke	9 (56.2%)	43 (78.2%)	
Brain tumor	7 (43.8%)	12 (21.8%)	
Behavioral assessments			
K-MMSE ~ 2	27.4 ± 2.6	28.0 ± 2.5	0.421
Trunk Control Test	84.6 ± 17.9	88.3 ± 15.0	0.459
FMA-UE (affected side)	53.6 ± 16.7	52.4 ± 14.9	0.798
Arm score of the Motricity Index (affected side)	86.3 ± 18.4	79.6 ± 21.0	0.225

Demographic and clinical characteristics of the calibration (pilot) cohort used to establish feedback thresholds. Values are reported as mean ± SD or n (%), as appropriate. K-MMSE ~ 2, Korean Mini-Mental State Examination ~ 2; FMA-UE, Fugl–Meyer Assessment for Upper Extremity

The remaining 55 participants constituted the main study cohort and were randomly assigned to the Non-feedback (NFB) group (n = 28) or the Feedback (FB) group (n = 27). The mean age of the cohort was 55.6 ± 16.6 years, with 28 males (50.9%) and 27 females (49.1%). The primary diagnosis was stroke (43/55, 78.2%), followed by brain tumor (12/55, 21.8%). Detailed demographic and baseline clinical characteristics are summarized in Table 2. Statistical analysis confirmed that there were no significant differences between the FB and NFB groups in terms of age, sex, affected side, diagnosis, or baseline clinical scores indicating that the two groups were well-balanced at baseline.

**Table 2:** Demographics and baseline clinical characteristics of the main study cohort by group

	Total (n = 55)	FB group (n = 27)	NFB group (n = 28)	*p*-value (FB vs. NFB)
Demographics				
Age (years)	55.6 ± 16.6	53.9 ± 17.4	57.3 ± 15.9	0.453
Gender, n (%)				1.000
Male	28 (50.9%)	14 (51.9%)	14 (50.0%)	
Female	27 (49.1%)	13 (48.1%)	14 (50.0%)	
Affected side, n (%)				0.422
Right	29 (52.7%)	11 (40.7%)	15 (53.6%)	
Left	26 (47.3%)	16 (59.3%)	13 (46.4%)	
Diagnosis, n (%)				0.528
Stroke	43 (78.2%)	20 (74.1%)	23 (82.1%)	
Brain tumor	12 (21.8%)	7 (25.9%)	5 (17.9%)	
Behavioral assessments				
K-MMSE ~ 2	28.0 ± 2.5	28.5 ± 2.6	27.6 ± 2.3	0.180
Trunk Control Test	88.3 ± 15.0	86.6 ± 16.3	90.0 ± 13.5	0.404
FMA-UE (affected side)	52.4 ± 14.9	51.5 ± 14.6	53.3 ± 15.2	0.656
Arm score of the Motricity Index (affected side)	79.6 ± 21.0	78.9 ± 22.7	80.3 ± 19.2	0.806

K-MMSE ~ 2, Korean Mini-Mental State Examination ~ 2; FMA-UE, Fugl–Meyer Assessment for Upper Extremity. FB, Feedback; NFB, Non-Feedback

A priori sample size estimation was performed using G*Power software (version 3.1) [47]. Based on previous rehabilitation intervention studies investigating feedback-based training targeting trunk compensation, which reported large intervention effects [48, 49], an effect size of 0.8 was assumed for the present study. Using a two-tailed independent samples t-test with a significance level of α = 0.05 and statistical power of 0.80, the required total sample size was calculated to be 52 participants (26 per group). The present study included 55 participants in the main cohort (27 in the FB group and 28 in the NFB group), thereby satisfying the required sample size for adequate statistical power.

Experimental sessions were conducted in the rehabilitation therapy room under the supervision of an occupational therapist. The study protocol was approved by the Institutional Review Board (IRB) of Samsung Medical Center (Approval No. 2023-11-105) and conducted in accordance with the Declaration of Helsinki. Written informed consent was obtained from all participants or their legal guardians prior to enrolment. The study was registered at ClinicalTrials.gov (NCT07397767).

### Experimental design

**Fig. 2:**
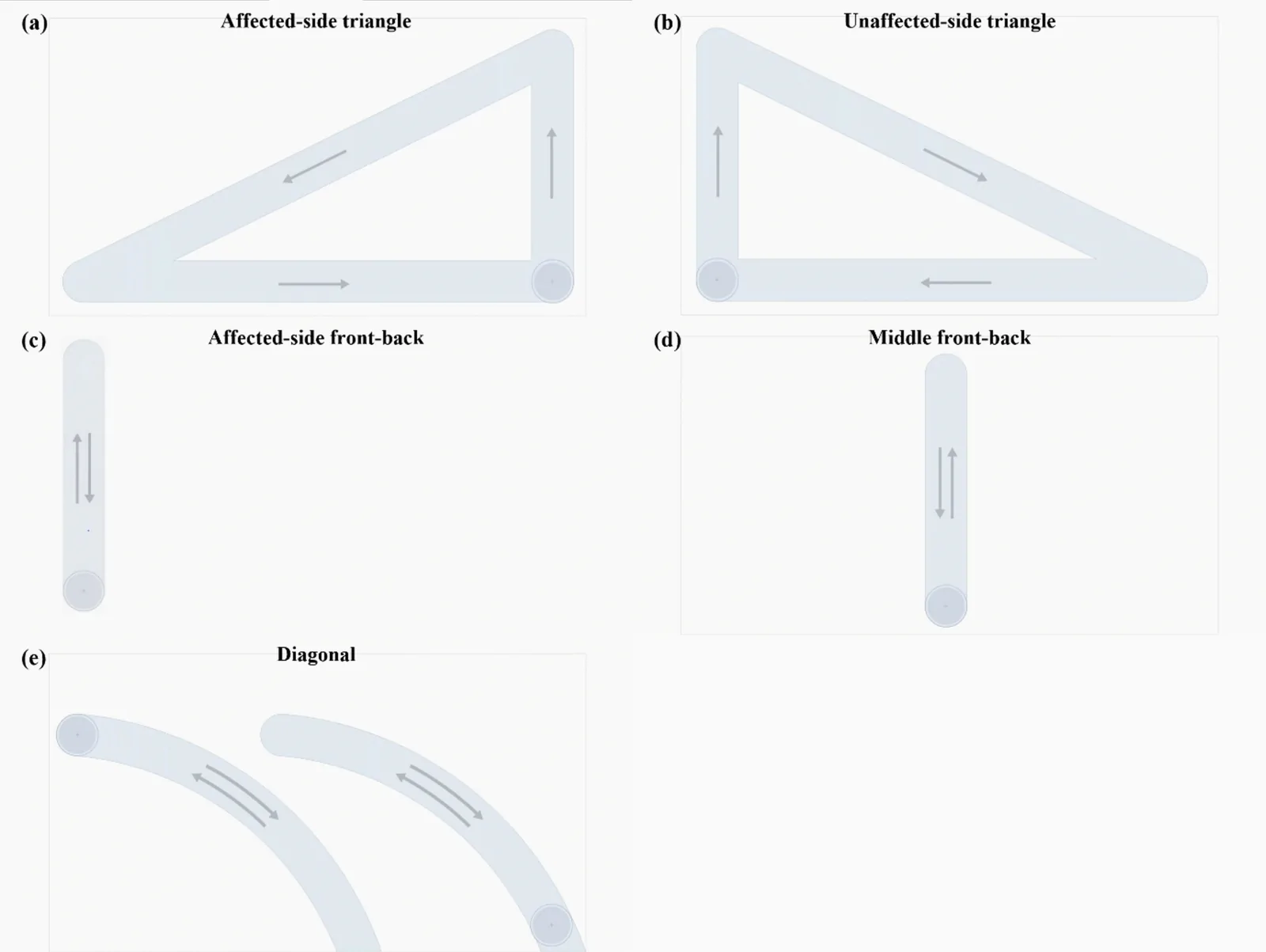
Smart board tasks. Five task protocols were administered: **a** affected-side triangle, **b** unaffected-side triangle, **c** affected-side front–back, **d** middle front–back, and **e** diagonal

Participants were seated in a chair or wheelchair in such a manner that they would be able to see the Smart Board. The Smart Board is a non-motorised, passive device in which participants grasp an open-type handle and slide it freely across a horizontal board surface without motorised assistance or physical arm support. In order to evaluate comprehensive upper limb function, five standardised tasks from the supervised upper extremity rehabilitation with the Smart Board (Fig. 2) were employed, using predefined digital templates to ensure consistent target positions and movement distances across participants; the same set of tasks was performed for both the affected and unaffected upper limbs, with each task repeated three times. These tasks were strategically selected to elicit key shoulder and elbow movement components corresponding to items in the Fugl–Meyer Assessment for Upper Extremity (FMA-UE) [50]. The specific tasks included the following drawing types: affected-side triangle, unaffected-side triangle, affected-side front–back, middle front–back, and diagonal drawings. The tasks were selected for their strategic value in eliciting key shoulder and elbow movement components corresponding to items in the FMA-UE. The triangle tasks (Tasks 1 and 2) require continuous changes in movement direction, with coordinated shoulder horizontal abduction/adduction and elbow flexion/extension. This reflects segmental control within synergy-like movement patterns. The anterior and posterior tasks (Tasks 3 and 4) primarily assess the ability to perform shoulder flexion while maintaining elbow extension. This approach enables the evaluation of voluntary movement that is less constrained by a pathological flexor synergy. Finally, the diagonal drawing task (Task 5) challenges the patient with complex multi-joint coordination across the transverse and sagittal planes, engaging coupled shoulder abduction and rotation. This task also approximates the advanced motor control required for movement combinations outside of basic synergies.

Participants were required to maintain a neutral upright posture for a period of 10 s with the objective of calibrating the system and establishing the reference coordinates (${n}_{ref}$). Subsequent to this calibration, participants performed each task on three occasions with both the affected and unaffected upper limbs. The FB group received real-time visual and auditory feedback whenever their trunk movement exceeded the pre-determined calibrated thresholds (Warning/Severe zones). The NFB group performed identical tasks, yet received no feedback. For the purpose of quantitative validation, it was necessary to undertake a simultaneous recording of all sessions. This was achieved by utilising an Intel RealSense D435i depth camera (Intel Corporation, USA) [51], which captures synchronized RGB and depth images. The depth data obtained from this device were used exclusively for post-hoc 3D kinematic reconstruction and ground-truth validation of trunk motion measurements (see the System Configuration and Feedback Algorithm section for further details).

### Behavioral assessments

Prior to the commencement of the session, all participants underwent evaluation of upper limb motor function using the FMA-UE, and the arm score of the Motricity Index [52] to assess upper limb motor power. The trunk control test [53] was utilised to evaluate trunk control function, and the K-MMSE ~ 2 was employed to assess cognitive function.

### System configuration and feedback algorithm

**Fig. 3:**
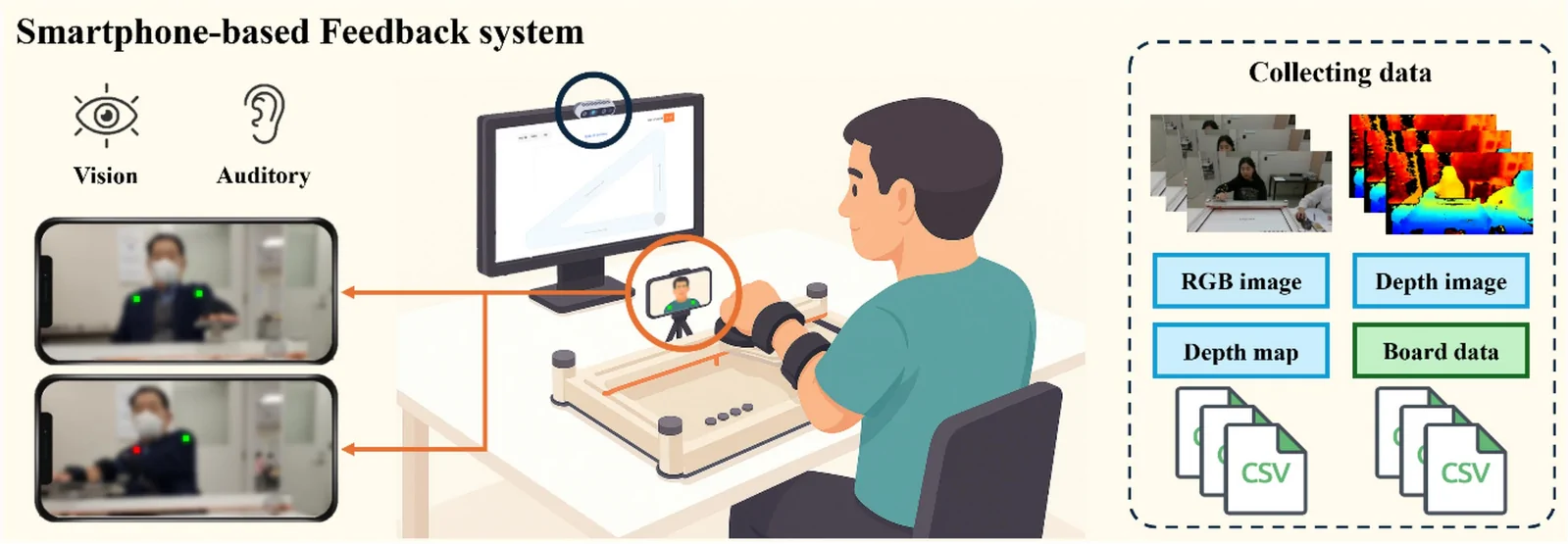
System overview. System overview of the proposed smartphone-based visual–auditory feedback system for trunk compensation monitoring during Smart Board upper limb tasks. RGB-Depth data and pose key points were used to reconstruct 3D kinematics and determine compensation severity, which was delivered to the user as real-time feedback

An interactive smartphone-based visual–auditory feedback system integrated with the Rapael Smart Board™ was developed to monitor and reduce excessive trunk movement during upper limb tasks. The system is comprised of a smartphone for real-time pose estimation and a Smart Board for the provision of task-oriented therapy exercises (Fig. 3). The smartphone-based monitoring system was implemented using a Samsung Galaxy Z Flip3 (Samsung Electronics, South Korea). The front-facing camera (10 MP, f/2.4 aperture, ~ 80° field of view) was used to capture RGB frames at up to 30 frames per second for real-time pose estimation. An Intel RealSense depth camera was utilised in parallel to record all sessions for the purpose of analysis of three-dimensional (3D) kinematics. The captured video frames were processed in real time on-device using MediaPipe Pose Landmarker in live-stream mode [38]. MediaPipe Pose has been widely validated in prior biomechanical studies for its reliable joint tracking [39, 54]. Therefore, it was employed in this system as a standard, validated modality to enable real-time feedback, allowing the study to focus on rehabilitation efficacy. To evaluate the agreement between the MediaPipe-based depth estimation and the reference depth measurements, Pearson correlation analysis was performed.

The real-time feedback pipeline comprised four sequential stages: (1) pose detection, in which the front-facing camera captures RGB frames at up to 30 fps and MediaPipe Pose Landmarker detects body landmarks on-device; (2) feature extraction, in which relative shoulder depth and reference-plane deviation angle are computed at each frame; (3) threshold evaluation, in which the extracted values are classified into one of three severity zones based on pre-calibrated thresholds; and (4) feedback generation, in which visual traffic-light overlays are rendered immediately on screen, and auditory alerts are triggered based on the accumulated severity state over a 5-s window [41, 55, 56].

In order to differentiate between normal motion and compensatory trunk movements, a feedback logic was established on the basis of two kinematic metrics derived from the 3D landmarks. In this study, compensatory trunk movement is defined as deviation of the trunk from a neutral reference posture established during the initial calibration phase. This definition is consistently applied across all analyses, and different kinematic metrics are used to quantify this deviation from complementary perspectives. Specifically, real-time detection of compensatory trunk movement is performed using frame-level kinematic metrics, including relative shoulder depth and deviation angle φ(t), which are used to classify posture severity and trigger feedback during task execution. In addition, trajectory-based metrics, such as total shoulder path length, spatial occupancy, and projected area, are used for post-hoc quantitative evaluation of compensatory trunk movement over the entire task duration. The first of these metrics is relative shoulder depth, which represents anterior–posterior trunk lean (flexion/extension). The second metric is reference-plane deviation angle, which represents the deviation of the shoulder vector from the neutral sitting plane. The deviation angle $\varphi \left(t\right)$ was calculated as follows:

1 $$\begin{array}{c}{v}_{shoulder}= {p}_{shoulder}- {p}_{center}\end{array}$$

2 $$\begin{array}{c}\varphi \left(t\right)=arccos\left(\frac{{v}_{shoulder}\left(t\right)\cdot {n}_{ref}}{\Vert {v}_{shoulder}\left(t\right)\Vert \Vert {n}_{ref}\Vert }\right)-90^\circ \end{array}$$

where ${v}_{shoulder}$ denotes the shoulder direction vector defined as the vector from the center point ${p}_{center}$ to the shoulder landmark ${p}_{shoulder}$. The center point ${p}_{center}$ is defined as the midpoint of the left and right shoulder joints obtained during the initial calibration posture. Here, ${p}_{LS}$ and ${p}_{RS}$ denote the 3D coordinates of the left and right shoulder landmarks obtained from the markerless pose estimation model, and ${p}_{shoulder}$ represents either ${p}_{LS}$ and ${p}_{RS}$. The vector ${n}_{ref}$ denotes the unit normal vector defined during the initial calibration posture, representing the neutral trunk orientation. The deviation angle *ϕ (t)* represents the angular difference between the current shoulder vector and the reference vector ${n}_{ref}$. This measure reflects a three-dimensional deviation magnitude from the neutral posture. Accordingly, it captures overall trunk displacement irrespective of movement direction.

To ensure the provision of objective feedback, thresholds for these metrics were pre-calibrated using a pilot dataset collected from 16 participants prior to the main experiment. The distribution of depth and angle values in the pilot cohort was analysed, and three severity zones were defined based on percentile ranges: The colour green indicates the normal range, defined as the 25th to 75th percentile; yellow indicates a warning level, defined as values between the 5th–25th and 75th–95th percentiles; and red indicates a severe level, defined as the 95th percentile. The specific threshold ranges are detailed in Table 3. At each frame, both metrics were independently classified into one of three severity zones based on their respective pre-calibrated thresholds derived from the pilot cohort and applied uniformly across all participants in the main study, and the overall severity zone was determined by the more severe of the two individual classifications. The system provides real-time visual cues in the form of traffic-light colours overlaid on the user’s screen image, along with auditory alerts triggered based on the accumulated severity state over a 5-s window to reduce excessive feedback frequency.

**Table 3:** Feedback threshold ranges for trunk compensation severity

Zone	Percentile range	Range of Z-value	Range of trunk angle
Normal	25–75%	[− 0.243, − 0.137]	[− 15.6118, 27.174]
Warning	5–25% 75–95%	[− 0.329, − 0.243], [− 0.137, − 0.035]	[− 36.514, − 15.611], [27.174, 59.616]
Severe	< 5%, > 95%	< − 0.329, > − 0.035	< − 36.514, > 59.616

The distribution of depth and angle values in this cohort was analysed, and three severity zones were defined based on percentile ranges: green indicates the normal range (25th–75th percentile); yellow indicates a warning level (5th–25th and 75th–95th percentiles); and red indicates a severe level (< 5th or > 95th percentile)

### Evaluation metrics

In order to validate the clinical efficacy and usability of the proposed system, the evaluation was conducted in two phases: a quantitative analysis and a usability assessment. The quantitative analysis comprised four categories of metrics: (1) trunk kinematics metrics, which characterised compensatory trunk movement patterns; (2) shoulder kinematics metrics, which quantified range-of-motion of the affected upper limb; (3) task performance metrics, which assessed hand movement efficiency derived from the Smart Board; and (4) behavioral response metrics (Feedback group only), which quantified immediate motor responses to the real-time feedback signals. The usability assessment evaluated user satisfaction and system feasibility through a structured questionnaire.

### Quantitative analysis

The quality of movement and compensatory patterns was evaluated through a comparison of the FB group and the NFB group during task execution. Prior to analysis, tracking artefacts were removed by excluding frames in which the visibility score of the shoulder landmark, as provided by the MediaPipe Pose model, fell below 0.5; these frames were marked as missing values and replaced via linear interpolation. The 3D point data *(X, Y, Z)* were reconstructed by integrating MediaPipe Pose landmark detection with the depth camera measurements. The 2D pixel coordinates $\left(u,v\right)$ of the landmarks detected by MediaPipe Pose model were used to sample the corresponding depth value from the synchronised depth map. The utilisation of the camera's intrinsic parameters, namely focal lengths ${f}_{x}, { f}_{y}$ and principal points ${c}_{x}, {c}_{y}$ facilitated the back-projection of these coordinates into metric three-dimensional space.

3 $$\begin{array}{c}Z(u, v)=D\left(u,v\right)\end{array}$$

4 $$\begin{array}{c}X=\frac{\left(u-{c}_{x}\right)}{{f}_{x}}\cdot Z, Y=\frac{\left(v-{c}_{y}\right)}{{f}_{y}}\cdot Z\end{array}$$

where *(u, v)* denotes the 2D landmark coordinates detected by the MediaPipe Pose model, and $D\left(u,v\right)$ represents the depth value at pixel coordinate $\left(u, v\right).$ Utilising these reconstructed 3D coordinates, we proceeded to compute shoulder range-of-motion and spatial dispersion metrics.

#### Trunk kinematics metrics

Compensatory trunk movement was quantified using the trajectory of the shoulder landmark. Specifically, the total accumulated displacement of the affected shoulder was computed to reflect trunk movement during task execution. A larger shoulder path length indicates greater trunk involvement, whereas a reduced path length reflects suppression of compensatory trunk motion.

5 $$\begin{array}{c}{L}_{shoulder}=\sum_{t=0}^{T-1}{\Vert P\left(t+1\right)-P\left(t\right)\Vert }_{2}\end{array}$$

The maximum excursion of trunk movement was quantified in both anterior and lateral directions. The maximum anterior excursion was defined as the maximum forward displacement relative to the initial position, and the maximum lateral excursion was defined as the maximum lateral displacement relative to the initial position during task execution, as follows:

6 $$\begin{array}{c}{E}_{anterior}=\underset{t}{\mathit{max}}\left|y\left(t\right)-y\left(0\right)\right|\end{array}$$

7 $$\begin{array}{c}{E}_{lateral}=\underset{t}{\mathit{max}}\left|x\left(t\right)-x\left(0\right)\right|\end{array}$$

In order to quantify the extent of motion within specific planes, the 3D shoulder trajectory was projected onto the XY (Transverse), YZ (Sagittal), and ZX (Coronal) planes. The convex hull area was then computed for each projection. In the *XY, YZ,* and *ZX* planes, the 3D trajectory of the affected side shoulder was projected, and 2D points (*x, y*) were obtained. The convex hull that is the smallest enclosing convex polygon of these points was computed and its area was calculated. In the event of the convex hull vertices being arranged in a counterclockwise sequence, that is to say, if ${\left\{\left({x}_{k},{y}_{k}\right)\right\}}_{k=1}^{K}$, then the area of the hull can be calculated using the so-called shoelace formula.

8 $$\begin{array}{c}\begin{array}{c}{A}^{P}= \frac{1}{2}\left|\sum_{k=1}^{K}\left({x}_{k}{y}_{k+1}- {x}_{k+1}{y}_{k}\right)\right|\\ \left({x}_{K+1},{y}_{K+1}\right)=\left({x}_{1},{y}_{1}\right) where P \in \left\{XY,YZ,ZX\right\}\end{array}\end{array}$$

A smaller ${A}^{P}$ indicates a more controlled and less variable trajectory within that plane.

In order to assess postural stability in the transverse (XY) plane, the area of the 95% confidence ellipse was calculated. This ellipse is known to capture the core region of trunk sway while excluding outliers.

9 $$\begin{array}{c}{CEA}_{95\%}=\pi \cdot {\chi}_{\mathrm{0.95,2}}^{2}\cdot \sqrt{{\lambda}_{1}{\lambda}_{2}}\end{array}$$

The volume of the axis-aligned bounding box (AABB) enclosing the entire affected-side shoulder trajectory was calculated. This metric is indicative of the total spatial occupancy, reflecting the extent of compensatory swaying.

10 $$\begin{array}{c}Volume= \left(\underset{t}{\mathit{max}}{X}_{t}-\underset{t}{\mathit{min}}{X}_{t}\right)\left(\underset{t}{\mathit{max}}{Y}_{t}-\underset{t}{\mathit{min}}{Y}_{t}\right)\left(\underset{t}{\mathit{max}}{Z}_{t}-\underset{t}{\mathit{min}}{Z}_{t}\right)\end{array}$$

#### Shoulder kinematics metrics

The angle of abduction and flexion of the shoulder was computed in the camera coordinate system. This was achieved by projecting the vector of the upper arm onto the camera XY plane (i.e. the image plane) and the camera YZ plane [42, 57]. The upper-arm vector is defined as:

$${P}_{sh} :3D coordinates with shoulder$$

$${P}_{el} :3D coordinates with elbow$$

11 $$\begin{array}{c}u\left(t\right)={P}_{el}\left(t\right)-{P}_{sh}\left(t\right)\end{array}$$

In order to obtain the elevation angles specific to each plane, the function u *(t)* was projected onto the corresponding planes. Shoulder abduction projected onto the camera XY plane:

12 $$\begin{array}{c}{\theta}_{abd}\left(t\right)=atan2\left(\left|{u}_{x}\left(t\right)\right|, \left|{u}_{y}\left(t\right)\right|\right)\cdot \frac{180}{\pi }\#\end{array}$$

shoulder flexion projected onto the camera YZ plane:

13 $$\begin{array}{c}{\theta}_{flex}\left(t\right)=atan2\left(\left|{u}_{z}\left(t\right)\right|, \left|{u}_{y}\left(t\right)\right|\right)\cdot \frac{180}{\pi }\#\end{array}$$

absolute value was used to report unsigned elevation magnitude (0–180°). *atan2* returns the angle in radians, which was converted to degrees by multiplying by *180/π *.

#### Task performance metrics

To evaluate functional upper limb performance independently of trunk movement, task performance metrics were derived from the Smart Board system. The hand trajectory, recorded directly from the board interface, was analysed to compute two primary parameters: (1) hand path length, defined as the cumulative distance travelled by the end-effector on the board surface, and (2) average speed, representing the mean velocity during task execution,, and (3) task duration, defined as the total time required to complete each task. These metrics reflect task execution efficiency rather than compensatory trunk movement.

#### Behavioral response metrics

For the FB group, the efficacy of the real-time feedback was further quantified by analysing immediate behavioural regulation. Traditional kinematic metrics may not fully capture the complexity of cognitive–motor interactions, particularly in distinguishing between reactive and proactive adjustments [58]. The present study therefore involved the development of three new responsiveness metrics based on state-transition logic. Key metrics included the correction, Red-to-Yellow-to-Green, warning response rates.

The correction rate is defined as the percentage of successful returns to the normal zone immediately following an alert. It functions as a primary metric of reactive compliance to external stimuli. The Red-to-Yellow-to-Green rate was measured as the ratio of corrections transitioning from Severe to Normal via the Warning zone. In contradistinction to other types of corrections, which have the potential to result in overshooting, this sequential transition reflects a gradual and controlled posture adjustment.

14 $$\begin{array}{c}Correction Rate= \frac{{N}_{RG}+{N}_{RY}}{{N}_{R}}\end{array}$$

15 $$\begin{array}{c}RYG rate= \frac{{N}_{RYG}}{{N}_{R}}\end{array}$$

where ${N}_{RG}$ and ${N}_{RY}$ represent the number of red-to-green and red-to-yellow state transitions, respectively, ${N}_{RYG}$ denotes the number of red–yellow–green transitions, and ${N}_{R}$ represents the total number of frames in the red state.

Statistical comparisons used the participant as the unit of analysis throughout. For between-group comparisons of across-task kinematic metrics, each participant's values were averaged across the five tasks to yield a single value per participant, compared using Welch's t-test (FB: n = 27; NFB: n = 28). For task-specific performance comparisons (Supplementary Table 1), each participant contributed a single value per task, averaged across the three repetitions, and the groups were compared within each task (FB: n = 27; NFB: n = 28). For within-subject comparisons between the affected and unaffected sides in the FB group, each participant contributed a single value per side, compared using the Wilcoxon signed-rank test (n = 27 pairs). All tests were two-tailed (significance level *p* < 0.05), and p-values were adjusted for multiple comparisons using the Benjamini–Hochberg false discovery rate (FDR) procedure.

### Usability assessment

In order to assess the internal consistency and reliability of the usability survey, Cronbach's alpha (*α *) was calculated. The survey evaluated user satisfaction, system clarity, and ease of use. Cronbach's alpha is defined as:

16 $$\begin{array}{c}\alpha =\frac{k}{k-1}\left(1-\frac{\sum_{i=1}^{k}{\sigma}_{i}^{2}}{{\sigma}_{T}^{2}}\right)\end{array}$$

where *k* is the number of survey items, ${\sigma}_{i}^{2}$ is the variance of the scores for the *i*-th item, and ${\sigma}_{T}^{2}$ is the variance of the total scores. Based on standard psychometric guidelines, an *α * coefficient ranging from 0.70 to 0.90 was considered indicative of acceptable internal consistency, while values exceeding 0.90 may suggest redundancy among items [59]. The full usability questionnaire is provided in Supplementary Table 2.

## Results

### Quantitative analysis

Prior to group comparison, the validity of the MediaPipe-based kinematic estimates was confirmed through post-hoc comparison with RealSense D435i measurements, yielding a depth trajectory correlation of r = 0.80 ± 0.25 (affected: r = 0.78 ± 0.27; unaffected: r = 0.82 ± 0.23; Supplementary Fig. 1) based on relative motion patterns, and a deviation angle estimation error of RMSE = 8.41 ± 7.62° and MAE = 7.81 ± 7.46°.

#### Clinical efficacy: suppression of trunk compensation

The quantitative analysis revealed significant between-group differences in trunk kinematics and spatial occupancy metrics.

As shown in Table 4, the FB group exhibited significantly higher postural stability than the NFB group. Specifically, the trunk path length was significantly shorter in the FB group (mean = 9.89 m) compared to the NFB group (mean = 15.91 m), representing a 37.9% reduction (*p* = 0.014). This indicates that participants receiving feedback completed the Smart Board tasks with substantially less unnecessary trunk excursion.

**Table 4:** Group comparison of key kinematic parameters

Metric	FB group (n = 27)	NFB group (n = 28)	Diff (%)	FDR-adjusted *p*-value
Path length	9.887 ± 5.923	15.910 ± 10.082	− 37.9^*^	0.014
(m)				
Bounding box vol	0.076 ± 0.048	0.117 ± 0.044	− 35.3^**^	0.003
(m^3^)				
Projected area XY	0.056 ± 0.032	0.097 ± 0.031	− 41.8^***^	< 0.001
(m^2^)				
95% confidence ellipse area (m^2^)	0.090 ± 0.050	0.142 ± 0.057	− 37.0^**^	0.002
Max excursion anterior	0.189 ± 0.052	0.224 ± 0.047	− 15.3^*^	0.015
(m)				
Max excursion lateral	0.265 ± 0.106	0.329 ± 0.079	− 19.4^*^	0.015
(m)				

Values are Mean ± SD. Diff (%) = (FB − NFB)/NFB × 100. Between-group comparisons used Welch's t-test on participant-level means (each participant averaged across five tasks), with FDR correction (Benjamini–Hochberg). *p < 0.05, **p < 0.01, ***p < 0.001

Spatial analysis further corroborated these findings. The bounding box volume, representing the total 3D space occupied by the shoulder movement, decreased by 35.3% in the FB group (0.076 ± 0.048 m^3^) compared to the NFB group (0.117 ± 0.044 m^3^; *p* = 0.003). Notably, the maximum excursion in the anterior direction, which is indicative of forward trunk lean, was significantly reduced in the FB group (0.189 m vs. 0.224 m; *p* = 0.015), confirming that the system effectively prevented compensatory forward leaning.

**Fig. 4:**
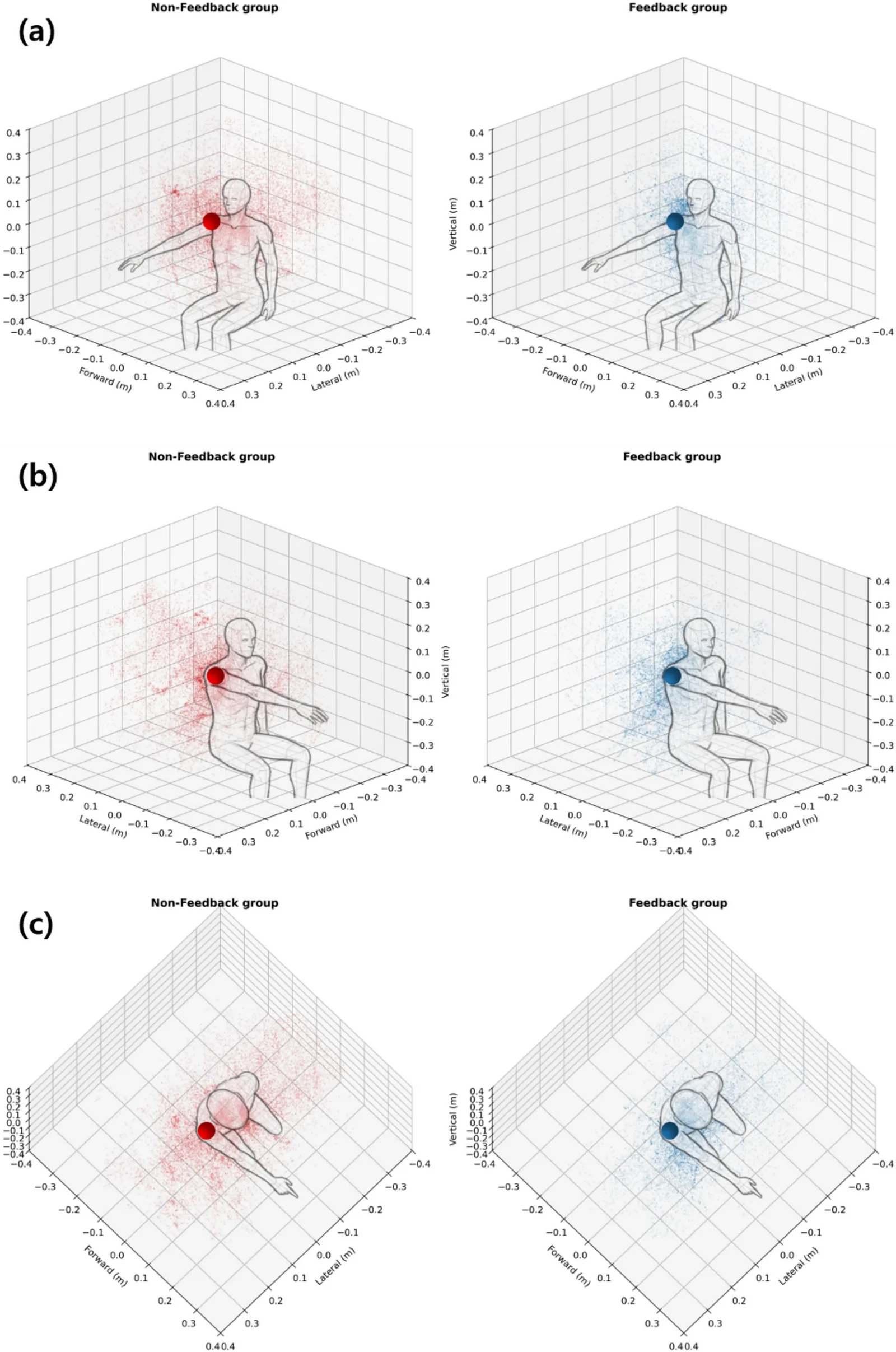
Spatial distribution of 3D accumulated shoulder trajectories representing deviations in the transverse, sagittal, and coronal planes. Red points in the NFB group form a widely dispersed cloud, illustrating significant anterior–lateral compensatory sway. In contrast, Blue points in the FB group show a tightly concentrated distribution near the neutral position, visually confirming the effective suppression of trunk compensation

**Fig. 5:**
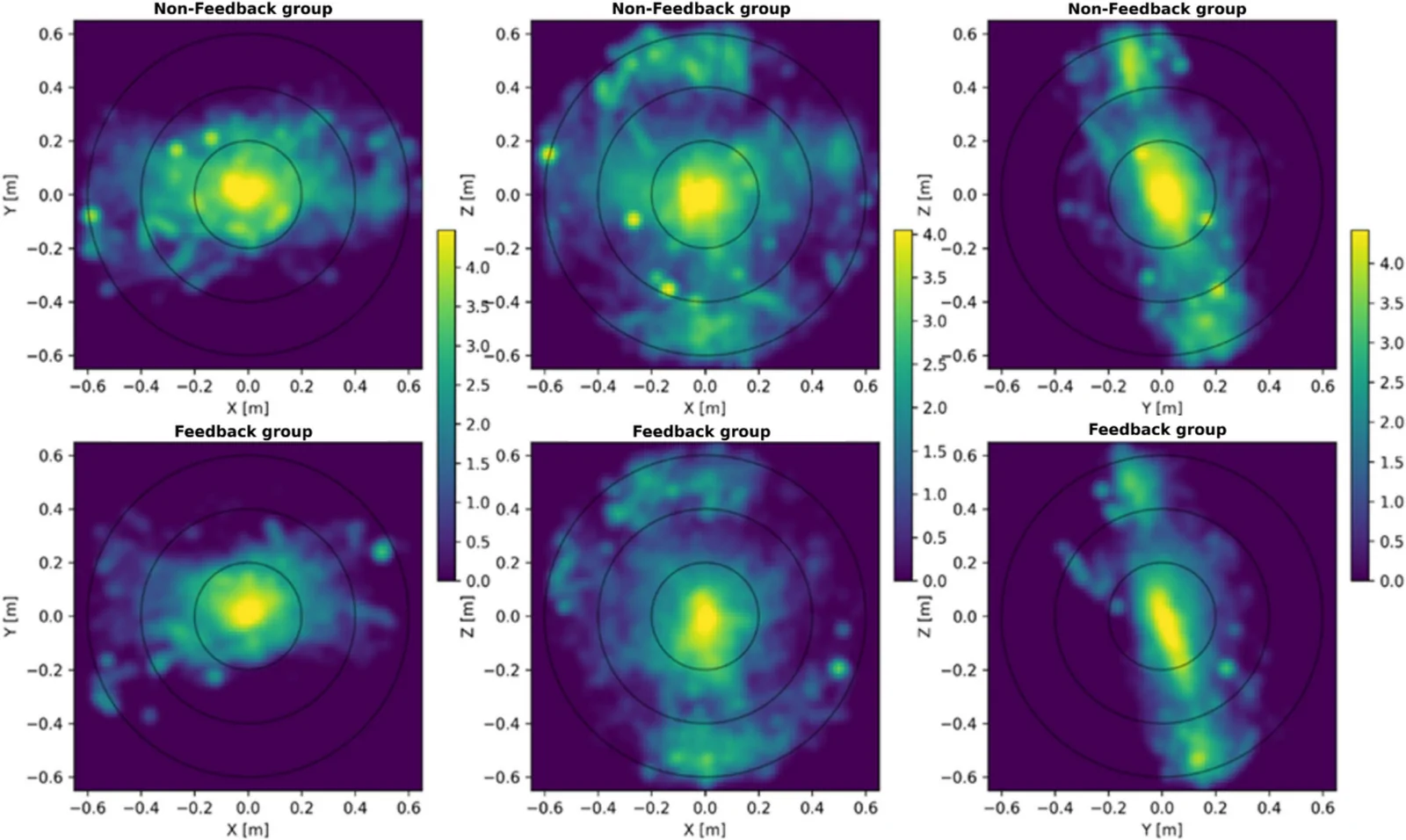
Spatial density maps of shoulder movement. Comparison of movement density in **a** transverse, **b** sagittal, and **c** coronal planes. The FB group demonstrates a highly concentrated distribution, visually corroborating the quantitative reductions in Projected Area (− 41.8%) and 95% Confidence Ellipse Area (− 37.0%)

The spatial density maps (Figs. 4 and 5) visually confirm these statistical differences. While the NFB group showed widely dispersed and irregular trajectories extending into anterior–lateral regions, the FB group exhibited constrained and tightly clustered patterns centered near the neutral position. Consequently, the projected movement area in the transverse (XY) plane was 41.8% lower in the FB group (0.056 m^2^) than in the NFB group (0.097 m^2^; *p* < 0.001). Similarly, the 95% Confidence Ellipse Area was reduced by 37.0% in the FB group (0.090 m^2^ vs. 0.142 m^2^; *p* = 0.002), indicating that feedback enabled patients to maintain their posture consistently within the optimal zone.

#### Impact on shoulder kinematics and task efficiency

Regarding shoulder range of motion (ROM), shoulder abduction was significantly reduced in the FB group (56.29° ± 15.17°) compared to the NFB group (64.36° ± 12.04°), corresponding to a 12.5% decrease (*p* < 0.05).

In terms of task performance (Supplementary Table 1), in Tasks 1–3, the FB group showed comparable or slightly longer hand path lengths, while demonstrating higher movement speeds and shorter task durations. This pattern suggests that the FB group's trajectories were executed more efficiently, reflecting more direct and goal-directed upper limb movement rather than compensatory trunk-assisted shortcuts. In Task 4, the FB group exhibited a slightly longer hand path length (1.035 m vs. 0.943 m, *p* < 0.05), with comparable movement speed and duration relative to the NFB group. In the more complex task (Task 5), the FB group achieved a shorter path length (3.305 m vs. 4.002 m, *p* < 0.01) and shorter duration (17.088 s vs. 29.833 s, *p* < 0.01), while movement speed was comparable between groups. These results suggest that whilst movements in the NFB group were slowed and prolonged by compensatory trunk strategies, the FB group achieved more efficient task execution despite engaging in less reliance on compensatory trunk-assisted movement strategies.

#### Behavioral response to feedback

To evaluate the immediate responsiveness to the system, we analyzed state-transition metrics within the FB group. Participants actively responded to the auditory and visual cues, even when using their affected side. Although the correction rate (successful return to the Normal zone after an alert) was lower on the affected side compared to the unaffected side (76.2% of the unaffected level; *p* = 0.032), the absolute rate (0.372) indicates that patients remained engaged and attempted active posture correction. Furthermore, the Red-to-Yellow-to-Green rate, which reflects controlled, gradual correction, was observed on the affected side, albeit at a lower frequency than on the unaffected side (*p* = 0.048).

### Usability assessment

Participants in the FB group completed a post-task survey assessing perceived usefulness and experience with the feedback system. The survey comprised three domains: overall usability/independent use (questions 1–2), feedback effectiveness and convenience (questions 3–7), and errors and improvement points (questions 8–9).The mean survey scores ranged from 3.44 to 4.07 on a 5-point Likert scale, indicating positive user acceptance. The survey responses were reliable, with an acceptable internal consistency (Cronbach’s α = 0.775, Supplementary Fig. 2). The highest average score was given to the question, “Was the feedback provided on mobile (visual and auditory) easy to understand and helpful?” (4.07), suggesting that the visual and auditory signals were easily understood. The second highest average score was for “Have you ever experienced the system disconnecting or failing to operate while in use?” (4.04), while the lowest score was for “Q1: Did you feel this rehabilitation feedback system was suitable for use by yourself?” (3.44). Detailed item-level results, including response distributions across all Likert-scale categories, are provided in Supplementary Table 3.

The usability evaluation confirmed high user acceptance, with participants recognizing the system's potential for independent use. Many participants specifically commented that the traffic-light–style visual feedback was intuitive, enabling them to recognize the target posture and task goals more easily. Nevertheless, qualitative feedback also revealed several practical limitations. Users reported difficulties with initial setup, particularly in aligning the starting position, and noted that the mobile device occasionally obstructed the Smart Board monitor during task performance. Additionally, several participants expressed a desire for supplementary quantitative numerical feedback alongside the traffic-light display to better track their performance progress over time.

## Discussion

The present study demonstrated that real-time smartphone-based visual-auditory feedback effectively suppressed compensatory trunk movements during upper limb rehabilitation tasks in hemiparetic patients. Whilst extant training systems principally concentrate on end-effector task performance, they often provide limited supervision over compensatory trunk strategies during upper limb rehabilitation [60, 61]. The proposed system addressed this gap by facilitating real-time monitoring of trunk compensation without necessitating wearable sensors or continuous therapist supervision.

The FB group exhibited a 37.9% reduction in trunk path length and a 35.3% decrease in spatial occupancy compared to the NFB group, indicating significant suppression of compensatory trunk movements. These findings indicate that the observed reduction reflects improved movement quality rather than simply reduced task execution. Notably, despite the NFB group attaining shorter hand path lengths on the Smart Board, kinematic analysis revealed that this apparent task efficiency reflected the utilization of kinematic redundancy—specifically, the recruitment of trunk degrees of freedom to minimize end-effector path length—rather than selective upper limb motor control. In contrast, the FB group demonstrated superior movement quality, maintaining trunk stability while engaging the shoulder and elbow joints independently, even though task performance metrics such as movement speed and duration were largely comparable between groups. These findings align with previous research demonstrating that without supervisory feedback, patients with hemiparesis tend to prioritise task completion over movement quality, relying on compensatory trunk strategies to compensate for limited arm function. Although some differences in shoulder kinematics may appear modest, it is important to interpret these values in the context of group-level analysis. These differences represent mean values averaged across participants, rather than single-frame measurements. As such, random frame-level pose estimation errors are likely to be attenuated through averaging, allowing systematic between-group differences to be detected. Therefore, even relatively small differences may reflect meaningful changes in movement patterns rather than measurement noise.

The suppression of compensatory trunk movements observed in the FB group is consistent with prior studies demonstrating the feasibility of automated compensation detection and feedback in stroke rehabilitation [12, 13, 41]. Previous approaches have shown that real-time feedback can reduce compensatory movements, while also emphasizing the importance of feedback timing and structure to avoid information overload [41]. In addition, studies using wearable sensor systems have highlighted the high prevalence of trunk compensation during functional tasks, underscoring the need for objective monitoring solutions [49]. Compared with previous studies utilising markerless systems to mitigate post-stroke compensation, the proposed system is distinguished by its clinical accessibility and scalability. Prior markerless systems have often relied on depth-sensing cameras, multi-camera setups, or laboratory-based motion capture environments [43], which may limit their scalability for unsupervised home use. In addition, Virtual Reality (VR)-based approaches developed to address trunk compensation [62] and VR-based educational interventions have shown improvements in postural control under structured conditions [63]. However, these approaches typically require specialised hardware or dedicated clinical setups. In contrast, the proposed system integrates real-time, threshold-based feedback to actively suppress compensatory trunk movements during task execution. By relying solely on a standard smartphone camera, it improves accessibility and scalability for unsupervised home use and provides quantitative evidence of reduced compensatory movement at the group level.

While the present study demonstrated the efficacy of the proposed system using the Rapael Smart Board™, the trunk compensation detection framework is not specific to this device and broadly applicable across diverse upper limb rehabilitation modalities. The system relies solely on a smartphone camera and MediaPipe-based pose estimation to track trunk kinematics, requiring no additional wearable sensors or device-specific integration. This architecture allows the system to be readily deployed alongside other planar or three-dimensional rehabilitation platforms, tabletop task-oriented training, mirror therapy setups, or even unstructured activities of daily living practice conducted at home. Given that compensatory trunk recruitment commonly occurs across virtually all upper limb rehabilitation tasks regardless of the specific device or task employed, the ability to monitor and suppress such compensation in a device-independent manner represents a meaningful advancement toward universal applicability. Future studies should investigate the performance of this system across varied rehabilitation tasks, patient populations, and clinical settings to further establish its generalizability and refine threshold calibration protocols for each modality.

Beyond its application as a standalone feedback tool, the proposed system holds considerable promise as a foundational technology for the emerging paradigm of the digital occupational therapist. In conventional rehabilitation environments, a therapist's ability to simultaneously monitor and correct compensatory trunk movements is limited by the one-to-one nature of in-person supervision. The present system, by automating real-time compensation detection and delivering structured visual-auditory feedback, effectively decouples the monitoring function from direct therapist presence. This creates a scalable infrastructure in which a single occupational therapist could remotely supervise multiple patients concurrently, forming a one-to-many rehabilitation monitoring model through a centralised dashboard that aggregates kinematic data streamed from each patient's smartphone. Such a model is particularly relevant in telerehabilitation settings, where geographic barriers, transportation limitations, and healthcare resource constraints frequently impede access to consistent, high-quality supervision. Furthermore, the objective kinematic metrics generated by the system, including trunk path length, spatial occupancy, and task efficiency, could serve as quantitative indicators that enable therapists to prioritize clinical attention toward patients exhibiting the most pronounced compensatory patterns, thereby optimizing the allocation of therapeutic resources. Collectively, these capabilities position the system not only as a feedback device, but as a core component of a digitally augmented occupational therapy practice capable of extending high-fidelity rehabilitation monitoring to large patient cohorts in unsupervised home environments.

The present study is subject to certain limitations that necessitate further investigation. First, the study was conducted at a single clinical center where the system was developed, which may introduce potential institutional or researcher bias. To mitigate this risk, the data analysis was conducted independently. In addition, several outcome measures exhibited relatively large variability, likely reflecting inter-individual differences in impairment severity and compensatory strategies among participants. Despite this variability, consistent group-level differences were observed across all measures. Future multi-center studies are required to further validate the generalizability of the findings.

Secondly, the study was conducted as a single-session cross-sectional observation, which limits the conclusions that can be drawn regarding long-term outcomes. It remains unclear whether the immediate corrective effects observed would be retained over time and translate into permanent motor learning, or whether patients would maintain consistent engagement with the system during repeated unsupervised home use. Patient adherence over extended periods is a critical determinant of rehabilitation outcomes and has yet to be formally evaluated. Furthermore, the long-term effects of sustained home use on upper limb functional recovery remain to be established. Accordingly, the present findings should be interpreted as preliminary evidence, and further longitudinal research assessing both adherence and clinical efficacy, including functional improvements in upper limb motor performance, is warranted prior to broader clinical implementation.

Thirdly, the current system utilised a fixed threshold for feedback triggers across all participants. Given the heterogeneity of hemiparesis, with patients exhibiting varying degrees of impairment, a universal criterion may not be applicable to all patients. It is recommended that future research efforts concentrate on the development of adaptive threshold algorithms that automatically personalise sensitivity in accordance with the individual's baseline capabilities.

Fourthly, the current system relies on a static calibration reference frame established at the start of each session. In unsupervised home settings, global shifts in sitting position relative to the camera may invalidate this fixed reference, potentially introducing false-positive or false-negative feedback. The system currently does not incorporate a mechanism to algorithmically distinguish between global body shifts and true trunk compensation. Future work should address this through the development of a dynamic recalibration module, including user-initiated recalibration, automatic periodic recalibration during inter-trial rest intervals, and pelvis landmark-based drift detection. In addition, the auditory feedback is based on a fixed time window and is not synchronized with task phases. Furthermore, more granular intra-task phase segmentation and waypoint-level target acquisition analysis may provide additional insights into movement strategies and task execution efficiency and should be incorporated in future studies.

Finally, the present study did not include pre-intervention baseline kinematic assessments. As the primary objective was to evaluate the immediate effect of real-time feedback on trunk compensation suppression during task execution within a single session, the study was not designed to measure individual-level functional change over time. While the randomized between-group design and the confirmed comparability of baseline clinical characteristics minimise the likelihood of systematic group differences, the absence of pre-intervention kinematic baselines limits the ability to fully exclude the contribution of pre-existing movement pattern differences to the observed outcomes. Future studies incorporating pre- and post-intervention kinematic assessments are warranted to evaluate the longitudinal effects of the feedback system on individual motor recovery.

## Conclusion

The clinical efficacy and usability of a mobile-integrated feedback system for the real-time detection and suppression of compensatory trunk movements during upper limb rehabilitation tasks was successfully validated. This study supports the clinical efficacy and usability of a mobile-integrated feedback system for the real-time detection and suppression of compensatory trunk movements during upper limb rehabilitation tasks. While the proposed system demonstrates strong potential for scalable and accessible rehabilitation, it currently relies on a fixed initial calibration and a time-based auditory feedback window. As a result, its ability to distinguish global body shifts from true trunk compensation and to provide phase-specific feedback may be influenced under certain unsupervised conditions. These aspects represent design considerations rather than fundamental limitations, and future work will focus on integrating dynamic recalibration and adaptive feedback strategies, along with AI-driven personalization, to further enhance robustness and enable reliable deployment in real-world home environments. It is anticipated that this technologically advanced apparatus will contribute to the standardisation of the quality of independent rehabilitation and the enhancement of functional outcomes for patients suffering from hemiparesis.

## Supplementary Information

Supplementary Material 1.

## Data Availability

The datasets used and analyzed during the study are available from the corresponding author on reasonable request.

## Abbreviations

3D: Three-dimensional
AABB: Axis-aligned bounding box
FB: Feedback
FMA-UE: Fugl–Meyer Assessment for Upper Extremity
IMUs: Inertial measurement units
IRB: Institutional Review Board
K-MMSE-2: Korean Mini-Mental State Examination-2
NFB: Non-Feedback
RGB: Red–Green–Blue
ROM: Range of Motion
VR: Virtual Reality
