# Supplementary material for: Smartphone-based real-time feedback to suppress trunk compensation for unsupervised upper limb rehabilitation in patients with brain disorders
Source: J Neuroeng Rehabil. 2026 Jul 16;23:302. doi: 10.1186/s12984-026-02093-5 (PMC13682079; doi:10.1186/s12984-026-02093-5)
Supplement: Supplementary file 1 [file 12984_2026_2093_MOESM1_ESM.docx]

**Supplementary Figure 1.** Distribution of Pearson correlation coefficients between MediaPipe-based and RealSense-based depth signals across all trials. Violin plots with embedded boxplots illustrate the distributions for overall, affected, and unaffected conditions.

**Supplementary Table 1. Smart Board Task Performance Metrics**

| Task | Length (m) | | Speed (m/s) | | Duration (s) | |
| --- | --- | --- | --- | --- | --- | --- |
|  | FB group | NFB group | FB group | NFB group | FB group | NFB group |
| Task1 (Affected Triangle) | 2.352 | 2.216 | 0.270 | 0.238 | 11.842 | 13.874 |
| Task2 (Unaffected Triangle) | 2.365 | 2.357 | 0.249 | 0.238 | 12.182 | 14.747 |
| Task3 (Affected Front-Back) | 0.956 | 0.916 | 0.208 | 0.179 | 5.259 | 6.983 |
| Task4 (Middle Front-Back) | 1.035* | 0.943 | 0.212 | 0.173 | 5.777 | 9.928 |
| Task5 (Diagonal) | 3.305** | 4.002 | 0.247 | 0.191 | 17.088** | 29.833 |

FB, Feedback; NFB, Non-Feedback. *p < 0.05, **p < 0.01, ***p < 0.001

**Supplementary Table 2. The usability assessment questionnaire**

**Usability Assessment Questionnaire**

*Smartphone-Based Real-Time Feedback System for Trunk Compensation Monitoring*

Please rate each item on a scale from 1 (Strongly Disagree) to 5 (Strongly Agree) by marking the appropriate circle.

For open-ended questions, please write your response in the space provided.

| **Name** |  | **Age** |  | **Sex** |  | **Date** |  | |
| --- | --- | --- | --- | --- | --- | --- | --- | --- |
| **No.** | **Item** | | | **Strongly agree** | **Agree** | **Neutral** | **Disagree** | **Strongly disagree** |
| **Usability of the Rehabilitation System** | | | | | | | | |
| 1 | Do you think the rehabilitation system is suitable for independent use? | | | ⑤ | ④ | ③ | ② | ① |
| 2 | Do you feel that the rehabilitation system was helpful for your treatment? | | | ⑤ | ④ | ③ | ② | ① |
| **Effectiveness and Convenience** | | | | | | | | |
| 3 | Was the feedback provided via the mobile device (visual and auditory) easy to understand and helpful? | | | ⑤ | ④ | ③ | ② | ① |
| 4 | Did you experience difficulty monitoring both the training screen and mobile feedback simultaneously during rehabilitation? | | | ⑤ | ④ | ③ | ② | ① |
| 5 | Did the posture feedback provided by the mobile system (visual and auditory) help you correct your posture during rehabilitation? | | | ⑤ | ④ | ③ | ② | ① |
| 6 | Do you feel that your motor ability or posture improved after using the rehabilitation system? | | | ⑤ | ④ | ③ | ② | ① |
| 7 | Did you find the mobile feedback system to be immediate and clear? | | | ⑤ | ④ | ③ | ② | ① |
| **Errors and Usability Issues** | | | | | | | | |
| 8 | Did you encounter any factors that caused mistakes while using the rehabilitation system? | | | ⑤ | ④ | ③ | ② | ① |
| 9 | Did you experience any interruptions or malfunctions while using the system? | | | ⑤ | ④ | ③ | ② | ① |
| 10 | If you felt any aspects of the rehabilitation system need improvement, please describe them. | | |  | | | | |
| **Applicability** | | | | | | | | |
| 11 | If this system were to be applied to other types of therapy, what kind of treatment do you think it would be suitable for? | | |  | | | | |
| **Open-Ended Questions** | | | | | | | | |
| 12 | Please provide any additional comments or suggestions. | | |  | | | | |

** This questionnaire was developed by the authors specifically for evaluating the usability of the smartphone-based trunk compensation feedback system used in this study.*

**Supplementary Figure 2. Average Survey Score per Question**

Mean usability survey scores (5-point Likert scale) for each question in the Feedback group.

**Supplementary Table 3. Item-level results of the usability assessment questionnaire (n = 27)**

| Item | Mean ± SD | Strongly Agree (5) | | Agree (4) | | Neutral (3) | | Disagree (2) | | Strongly Disagree (1) | |
| --- | --- | --- | --- | --- | --- | --- | --- | --- | --- | --- | --- |
| Q1 | 3.44 ± 1.12 | 18.5% | 37.0% | | 14.8% | | 29.6% | | 0.0% | |  |
| Q2 | 3.81 ± 0.68 | 11.1% | 63.0% | | 22.2% | | 3.7% | | 0.0% | |  |
| Q3 | 4.07 ± 0.62 | 18.5% | 74.1% | | 3.7% | | 3.7% | | 0.0% | |  |
| Q4 | 3.56 ± 1.09 | 14.8% | 55.6% | | 0.0% | | 29.6% | | 0.0% | |  |
| Q5 | 3.52 ± 1.01 | 14.8% | 44.4% | | 18.5% | | 22.2% | | 0.0% | |  |
| Q6 | 3.52 ± 0.98 | 14.8% | 40.7% | | 25.9% | | 18.5% | | 0.0% | |  |
| Q7 | 3.96 ± 0.59 | 11.1% | 77.8% | | 7.4% | | 3.7% | | 0.0% | |  |
| Q8 | 3.63 ± 0.97 | 11.1% | 63.0% | | 3.7% | | 22.2% | | 0.0% | |  |
| Q9 | 4.04 ± 0.85 | 25.9% | 63.0% | | 0.0% | | 11.1% | | 0.0% | |  |

Values are presented as mean ± standard deviation. Percentages indicate the proportion of responses for each Likert scale category (5 = strongly agree to 1 = strongly disagree).
